# A Retrospective Study on Prevalence and Surgical Management of Umbilical Hernias in Calves, 1998–2020

**DOI:** 10.1155/vmi/8838445

**Published:** 2025-04-14

**Authors:** Razieh Torkaman, Saeed Azizi, Ghader Jalilzadeh-Amin

**Affiliations:** ^1^Department of Surgery and Diagnostic Imaging, Faculty of Veterinary Medicine, Urmia University, Urmia, Iran; ^2^Department of Internal Medicine and Clinical Pathology, Faculty of Veterinary Medicine, Urmia University, Urmia, Iran

**Keywords:** calves, prevalence, surgical management, umbilical hernia

## Abstract

**Objective:** To investigate the prevalence and surgical treatment of umbilical hernias in calves.

**Animals:** Two hundred and sixty-seven calves with umbilical hernias.

**Methods:** Medical records of 505 calves with umbilical disorders between March 1998 and July 2020 were reviewed, with a specific focus on 267 calves diagnosed with umbilical hernias. Data on husbandry type, breed, gender, age, surgical techniques, and surgical outcomes were surveyed in this retrospective clinical study.

**Results:** The prevalence of umbilical hernia was 13.15% with simple umbilical hernias as the most commonly observed pathology. The number of calves with simple umbilical hernias was significantly higher than those with complicated hernias. Calves with umbilical infections exhibited a significantly higher prevalence of hernias (15.32%) compared to those without infection (9.26%). No significant differences were observed in the prevalence of hernias between breeds and genders. The frequency of hernias in calves managed under the range system was significantly higher than those managed under the smallholder system. Recurrence rates of hernias showed no significant differences between open and closed herniorrhaphy methods for the surgical treatment of simple umbilical hernias. 75.6% of calves with hernias were under 14 weeks of age, and the prevalence was almost nearly equal between females and males.

**Conclusion:** Umbilical hernias were more prevalent among Holstein calves raised under the range system with no observed sex predilection. Calves with umbilical infections were 1.77 times more likely to develop umbilical hernias compared to those without infections. No significant difference was found between open and closed methods of herniorrhaphy of simple umbilical hernias.

## 1. Introduction

Umbilical disorders are a frequent concern following birth in neonatal calves [1]. These disorders encompass umbilical herniation, infection, or a combination of both [2]. Enlargement of the umbilicus region is a common clinical sign. Umbilical hernias are among the most prevalent congenital defects in bovines with reported incidences ranging from 0.65% to 1.8% [3, 4]. Notably, Holstein and Holstein-Friesian breeds exhibit a considerably higher incidence [3, 5]. Umbilical hernias, with a crude risk of 15.1%, rank as the third most common cause of disease in dairy heifers under 3 months of age [6]. Therefore, the related economic impact on dairy farming including treatment costs, the loss of calves in breeding animals, and animal welfare issues should also be taken into consideration. The etiology of umbilical hernias remains under debate with theories suggesting either an inherited predisposition, generally accepted [5], and/or environmental factors such as umbilical infections [4, 5].

Umbilical hernias can be categorized as either simple/uncomplicated or complicated. Most hernias present as simple, nonstrangulating, and easily reducible [7]. However, the complicated ones can arise from concurrent subcutaneous abscesses or concurrent infections of the umbilical cord remnants with incomplete reducibility [5]. In rare instances, strangulating hernias may develop due to the entrapment of abomasum, small intestine, or greater omentum within the hernial sac [8]. Diagnosis of umbilical hernias typically relies on signalment and physical examination to assess the presence and reducibility of contents within a complete or incomplete hernial ring [5]. Ultrasonography is a valuable tool for precise diagnosis of the underlying pathology associated with the hernia [9, 10].

Treatment options for umbilical hernias encompass various techniques [8, 9, 11, 12]. Closed herniorrhaphy may be considered for uncomplicated hernias, and open herniorrhaphy is generally the preferred approach for most cases [5, 8]. The occurrence of umbilical hernias appears to be influenced by factors such as cattle breed, husbandry practices, and postpartum care provided to neonates [5]. This long-term retrospective clinical study aimed to investigate the prevalence of umbilical hernias in relation to husbandry type, breed, gender, age, surgical techniques employed, and surgical outcomes in calves with hernia.

## 2. Materials and Methods

### 2.1. Animals and Data Collection

This retrospective study reviewed the medical records of 295 calves admitted to the Veterinary Teaching Hospital, Faculty of Veterinary Medicine, Urmia University, Urmia, Iran, for the treatment of umbilical hernia between March 1998 and July 2020. The inclusion criteria were calves with umbilical mass/disorder, and the exclusion criteria were calves with no hernia. Therefore, out of 505 records, 267 records were included in the study. The study procedures were reviewed and approved by the Animal Ethics Committee of Urmia University (Ref No. IR-UU-AEC-3/86), and written consent was obtained from the animal owners. The calves were managed under either range conditions or in smallholder semi-industrial (free stall intensive) systems. The data retrieved from the medical records for analyses were husbandry type categorized as either range condition or smallholder semi-industrial system, breed, sex, and age of the calves. Also, details of clinical and physical examinations like the hernial ring palpation and reducibility of contents, surgical techniques, and outcomes were obtained.

### 2.2. Clinical Examinations and Systemic Medical Supportive Cares

Upon admission, all calves underwent a thorough physical examination. Diagnosis of umbilical hernias was primarily based on a combination of history, clinical, and ultrasonographical findings. In history taking information regarding the presenting signs, duration, and any relevant background, details were collected from the owner. The clinical examinations included a thorough inspection and palpation of the umbilical region in both standing and dorsal recumbency positions to assess the size of the hernial ring, reducibility of the content, and presence of inflammation or pain in the calves. In complicated cases, transabdominal ultrasonographic assessment was performed as a reliable diagnostic tool to identify concurrent underlying intraabdominal umbilical remnant pathologies (e.g., umbilical vein abscess) and to determine the optimal surgical approach for treatment, if necessary [10].

Based on the diagnosis (simple vs. complicated hernia), calves were assigned to one of two medical treatment approaches before the surgical intervention. The calves with hernia and concurrent umbilical pathology including concurrent umbilical abscess, enlarged umbilical stalk, and presence of local fistulas or sinus/drainage tracts received a preoperative systemic medical supportive care using the administration of antimicrobials and vitamins upon admission. Either a combination of procaine penicillin (22,000 IU/kg) and dihydrostreptomycin HCl (11 mg/kg) or oxytetracycline (10 mg/kg) was administered intramuscularly (IM) once a day for three consecutive days before surgical intervention. The calves received a single dose of vitamin AD_3_EC compound (3–6 mL/calf according to the manufacture's instruction) IM. The calves with simple (uncomplicated) hernias just received a prophylactic single dose of either procaine penicillin, dihydrostreptomycin HCl, or oxytetracycline 2 h before herniorrhaphy.

### 2.3. Anesthesia and Surgical Techniques

Calves with complicated umbilical hernias received intravenous (IV) administration of warmed isotonic crystalloid solutions such as normal saline or Ringer's solution, at a rate of 6–80 mL/kg/h as preoperative supportive care. This fluid therapy helped maintain hydration and electrolyte balance, particularly important in animals with potential dehydration or compromised circulation. For sedation, xylazine hydrochloride (0.1 mg/kg body weight) was administered IM. Calves were positioned in lateral or semidorsal recumbency on a surgical table during the surgical procedure. The ventral aspect of the abdomen was clipped, and the skin and abdominal wall surrounding the hernial sac were infiltrated with 2% lidocaine hydrochloride using a ring block technique. The surgical site was then scrubbed using povidone–iodine solution.

A detailed description of the umbilical herniorrhaphy techniques can be found elsewhere [1, 5, 8].

Briefly, an elliptical incision was made through the skin, centered on the hernial sac. A combination of blunt and sharp dissections was used on subcutaneous tissue to expose the hernial ring. In the closed technique, the hernial sac was inverted into the abdomen while keeping the peritoneum intact. In the open technique, the peritoneum was incised and the abdominal cavity was explored by digital palpation to identify any adherences or to determine the direction of infected remnants. The abdominal incision was extended to remove infected remnants and to separate adhesion, if necessary. In both techniques, the hernial defect was repaired using nonabsorbable #1 monofilament nylon (Supa, Karaj, Iran) suture in a series of modified Mayo overlapping sutures. The subcutaneous tissue was closed using absorbable suture, #1 chromic catgut, or Vicryl (Supa, Karaj, Iran), in a simple continuous pattern. The skin was closed with the same nonabsorbable #1 monofilament nylon sutures with a combination of interrupted vertical mattress and cruciate patterns.

### 2.4. Surgical Technique Selection and Management of Concurrent Umbilical Pathology

The selection of surgical technique for umbilical herniorrhaphy was chosen based on the size of the hernial ring and the presence of concurrent umbilical pathology. The technique of closed herniorrhaphy was chosen for uncomplicated hernias with a hernial ring less than 5 cm in diameter. The peritoneum remained intact during this procedure. However, open herniorrhaphy, which offers a greater access and visualization for addressing complicated hernias, was employed in hernias with a ring diameter exceeding 5 cm or with any evidence of local umbilical pathology regardless of the ring size [8].

Calves with umbilical hernias and concurrent umbilical abscesses/subcutaneous infections received one of the two treatment approaches: (1) Surgical intervention following conservative management or (2) direct surgical extirpation. In the first method, at first, a stab incision was created to allow drainage and facilitate curettage of the abscess cavity. Then, the abscess cavity was flushed with a diluted 0.05%–0.1% povidone–iodine solution to promote disinfection. Finally, the open herniorrhaphy to repair the hernial ring was typically performed 4–10 days after abscess drainage had stopped. In the second method, simultaneous surgical removal of the abscess and repair of the hernial ring were performed using the open herniorrhaphy technique [8].

Calves with complicated umbilical hernias including those with concurrent chronic active omphalitis, enlarged umbilical stalk, umbilical cord remnant infections, and fistulas received a two-stage treatment plan: As described previously, the calves underwent a preoperative medical regimen involving systemic antimicrobials and vitamins upon admission. Then, surgery was performed 3–5 days later via opening the hernial ring through an extension of the ventral midline incision [8] and the infected umbilical remnant(s) were removed.

### 2.5. Postsurgical Care and Follow-Up Evaluations

Following surgical repair, the calves received procaine penicillin plus dihydrostreptomycin HCl or oxytetracycline as mentioned before ones a day for 3–5 consecutive days accordingly. A single dose of vitamin AD_3_EC was administered, and the surgical skin wound was irrigated with povidone–iodine for 5–7 days. Owners recommended to limit the calves in stable/box for 4 weeks for recovery and skin sutures were removed 14 days after surgery. Follow-up information was obtained 2–4 weeks after surgery to determine surgical probable complications such as suture infections/abscesses, seromas, hematomas, dehiscence, recurrence of the hernia, local subcutaneous infection, and peritonitis. All recovered male calves with umbilical hernia were recommended to be raised for fattening.

### 2.6. Statistical Analysis

The chi-square test was used to compare the proportion of umbilical hernias between open and closed surgical techniques, as well as between breeds and genders in the calves. Furthermore, to assess the association between umbilical hernia and umbilical infection, a chi-square test was employed to compare the proportion of umbilical hernia in calves with and without umbilical infection. The strength of the association was further evaluated by calculating odds ratios (ORs) and its corresponding 95% confidence interval (CI). A statistically significant association was defined as a *p* value less than 0.05. All statistical analyses were performed using SPSS software (Version 27.0; SPSS Inc., Chicago, IL, USA).

## 3. Results

The overall prevalence of umbilical disorders was estimated at 22.50%, 505 out of 2244 cases. The prevalence of umbilical hernia (295/2244, 13.15%) was higher than those of umbilical remnant infection (210/2244, 9.36%). The general descriptive analysis of the collected data on umbilical hernia revealed that 267 calves were affected with umbilical hernia, 6 calves experienced intestinal evisceration through the umbilical opening, and 5 calves had a unique abdominal hernia located in the caudal portion of the intact umbilical ring.

Table 1 represents the frequency of simple and complicated umbilical hernias in relation to breed, sex, and age in the affected calves. The number of calves with simple umbilical hernia was higher than those with complicated umbilical hernias accounting for 229 out of 267 cases (85.77%) compared to 38 out of 267 cases (14.23%), respectively. The concurrent infections associated with complicated hernias included distinct pathologies like umbilical abscesses, enlarged stalk, infections of urachus, umbilical vein, paired umbilical arteries (either separately or concurrently), and draining fistulae.

Calves with umbilical infections exhibited a statistically significant (*p*=0.002) higher prevalence of umbilical hernias (15.32%) compared to calves without umbilical infection (9.26%). This association was further strengthened by OR analysis, revealing that calves with umbilical infections were 1.77 times more likely to develop an umbilical hernia (OR = 1.77; 95% CI: 1.22–2.57; *p*=0.002) compared to their infection-free counterparts (Table 2). These findings suggested a potential causal link between umbilical infection and subsequent hernia formation in calves.

**Figure 1:**
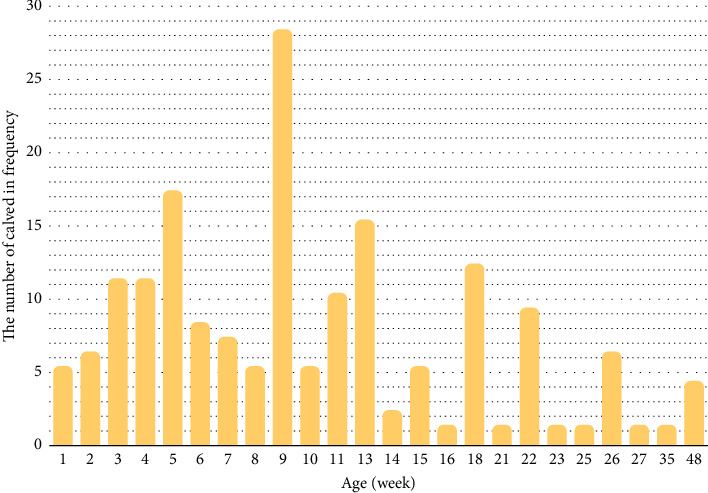
Distribution of umbilical hernia in calves according to the age (weeks).

Figure 1 displays the frequency of umbilical hernia in relation to the age of the calves. The analysis revealed that the age of the calves with hernia ranged from 2 weeks to 48 weeks with a median age of 9 weeks. Notably, 202 out of 267 calves with hernia (75.6%) were less than 14 weeks of age. Otherwise, the frequency of umbilical hernia was higher in calves during the second month of age suggesting a greater prevalence of hernia in younger calves that potentially was decreased with age.

The frequency of hernia was nearly identical between male (134/267, 50.19%) and female calves (133/267, 49.81%) (Table 1). There were no significant differences (*p*=0.21) in the prevalence of hernia between breeds and genders. The frequency of simple umbilical hernia was higher (229/267, 85.77%) compared to complicated umbilical hernia (38/267, 14.23%). Hernias were most prevalent in Holstein calves (229/267, 85.77%) compared to Native calves (27/267, 10.11%) and other breeds (11/267, 4.12%). The frequency of hernia in calves managed under the range system was significantly higher (255/267, 95.51%) to those managed under the smallholder system (12/267, 4.49%). The prevalence of simple hernias was also higher in calves raised in an extensive range system (220/229, 96.07%) compared to those raised in a smallholder system (9/229, 3.93%). Similarly, the prevalence of complicated hernias was greater in calves raised in the range system (35/38, 92.11%) than in those raised under the smallholder system (3/38, 7.89%).

Surgical herniorrhaphy proved to be highly successful in managing simple hernias. Recurrence rates were low, with only 8 out of 229 cases (3.49%) experiencing hernial recurrence. Notably, a second surgical intervention was successful in seven cases and culling was recommended for the one remaining unsuccessful case. There were no significant differences (*p*=0.85) in the recurrence rates of hernias between the open (6/143) and closed (2/76) methods of herniorrhaphy for the surgical treatment of simple umbilical hernias.

The analysis of complicated umbilical hernia in 38 calves showed that the prevalence of hernia with concurrent umbilical abscesses was higher (20/38, 52.63%) compared to hernia with an enlarged umbilical stalk (9/38, 23.68%) and hernia with infection of umbilical remnants (5/38, 13.16%) (Table 1). Almost all affected calves were Holsteins that raised in an extensive range management system (35/38, 92.11%). Regular antiseptic dipping of the umbilical cord following parturition was not a routine practice in the calves. Navel dipping was recorded just in two cases, and navel stalk ligation and cutting without dipping were recorded in four cases. Open herniorrhaphy was successful with some exceptions (recurrence of hernia in one calf and a subcutaneous abscess formation in one calf). One male Holstein calf presented with concurrent congenital partial spiral colon aplasia recommended for culling. Two other male Holstein calves with concurrent abomasal fistula were successfully treated with open herniorrhaphy. These calves were kept in a range system.

Six neonatal calves (various breeds: 1 male and 3 female Holsteins, 2 female Natives) were presented with a life-threatening condition of evisceration of gastrointestinal viscera through the umbilical opening. All the calves were kept in the range system. Surgical intervention resulted in a favorable outcome for two calves. However, the remaining four calves were in septic shock and were recommended for culling.

Five calves (4 Holstein and 1 Simmental) displayed an exceptional type of abdominal hernia with a similar appearance to the umbilical hernia but located more caudally to the intact umbilical ring. No history of trauma was identified in these calves, and no abnormalities were detected during clinical examinations or systematic exploration of the abdominal cavity. This finding suggested a potential congenital origin. Open herniorrhaphy effectively addressed this unique hernia type with no reported postoperative complications. All these calves were kept under range husbandry management.

## 4. Discussion

This long-term retrospective study analyzed medical records from a hospital setting over 22 years. The findings revealed a higher prevalence of umbilical hernias compared to umbilical remnant infections. These results were consistent with a similar long-term retrospective study conducted by Spadola et al. investigating umbilical disorders in calves. Their study reported a predominance of umbilical hernia (265/320, 82.81%) compared to purulent omphalitis (55/320, 17.19%) [13]. While both investigations adopted a retrospective design and a long-term timeframe, discrepancies in the prevalence of umbilical disorders could arise from differences in animal breeds, housing conditions, and husbandry practices. Additionally, the study methodology and different sample sizes appeared to play critical roles in influencing the outcomes.

The present study further differentiated simple and complicated umbilical hernias, revealing a dominance of simple hernias (229/267, 85.77%) compared to complicated cases (38/267, 14.23%). Spadola et al. (2022) reported that the prevalence of simple hernias was 65% (208/320) and that of complicated hernias was 35% (57/320) [13]. These variations potentially reflect differences in the level of health management practices employed in the respective farming systems.

Studies report varying rates of umbilical hernias among calves affected by umbilical disorders.

In the present study, the rate of the umbilical hernia was recorded to be 55.97%. Referral hospitals lead to a higher rate (63%) [14] or a lower rate (45%) [15] of the disorder. Farm management likely plays a role, so that intensive/semiextensive farms [13] showed a higher rate (82.81%) compared to our study (55.97%). The variations might stem from study design, breed inclusion, farm practices, and calf health protocols.

The current study showed that umbilical hernias in calves ranged from 2 to 48 weeks of age. However, a significantly higher prevalence was identified in calves under 14 weeks of age. This finding was consistent with prior prospective cohort studies by Virtala et al. who reported that 15.1% of dairy heifer calves exhibited umbilical hernia during the first trimester of life [6]. Similarly, Spadola et al. noted a high proportion (65.62%) of affected calves within the first 12 weeks of life [13]. These findings collectively suggested a markedly elevated risk of umbilical hernias in younger calves.

Published reports on the age of diagnosis for umbilical hernia in calves vary, ranging from 3 to 8 weeks of age [4]. The present study showed a median age of 9 weeks of age with the majority of diagnoses occurring within the first 3 months of life. This finding was in agreement with prior research by others [6, 14].

Early intervention for the management of umbilical hernia is critical. Surgical repair becomes increasingly challenging in calves exceeding 5–6 months of age [5, 16, 17]. Therefore, surgical correction is generally recommended for all calves exhibiting progressive enlargement of a simple umbilical hernia. Conversely, surgical intervention for complicated hernias should be postponed after appropriate resolution of any concurrent infection within the umbilical ring and surrounding tissues [5]. In general, the present study suggested that the majority of umbilical hernias, at least in Holstein and Holstein-cross calves, were likely diagnosed within the first 3 months of life, although their occurrence might have extended up to 48 weeks after birth. Early diagnosis and prompt surgical intervention for the hernias are crucial for optimal treatment outcomes [5, 17].

Reportedly, the incidence of congenital umbilical hernia was higher in males (2.2%) than females (1.5%) [4, 18]. The present study showed almost equal prevalence of umbilical hernia in female and male calves. In the other words, no significant differences were found in prevalence of hernia between breeds and genders. This finding contrasted with prior investigations suggesting a potential sex bias toward females [8, 13, 19]. While a genetic predisposition for umbilical hernias in Holstein breeds is suspected [11], the exact mode of inheritance remains unclear. The sex-linked hypothesis lacks conclusive evidence, and alternative models involving incomplete penetrance of dominant genes or environmental influences are plausible [4, 17]. These observed discrepancies in sex distribution across various studies warrant further investigation to definitively elucidate the role of sex in umbilical hernia development among calves. A comprehensive understanding of this association will contribute to improved preventive and management strategies for this condition.

In the present study, the hernia was more prevalent in Holstein than other breeds. These findings were in agreement with other studies that showed umbilical hernias were commonly recognized in dairy heifers [4] and less prevalent in beef cattle [5, 12]. In the present study, the prevalence of umbilical hernia was 13.15% in calves in a hospital-based population. In this study, the data collected from 53,105 calves from 77 livestock markets within a 2-year period showed the overall incidence of 1.8% for the congenital hernia [18]. This controversy may be resulted basically from the study design, referral hospital population versus livestock markets, and different enclosed breed calves in the investigations.

In the present study, the frequency of the simple hernia was higher than the complicated hernia and it was concluded that most of the hernias could have had a congenital origin. Umbilical infections are a well-documented risk factor for the development of umbilical hernias in calves [4, 20]. Existing literature reports concurrent infections in varying proportions ranging from 18.32% to 45% in calves with hernias [5, 21, 22]. While the present study identified a lower prevalence of concurrent infections (14.23%), it nonetheless reinforces the potential association between these conditions. Furthermore, this study revealed a statistically significant association between umbilical infections and increased odds of developing a hernia (1.8 times higher risk). This finding was consistent with a prior case–control study by Steenholdt and Hernandez which demonstrated a 5.65-fold greater risk of hernia in heifers with umbilical infections [20]. Notably, their study also estimated that preventing umbilical infections could have potentially reduced the incidence of hernias in Holstein heifers by 82% [20]. Despite genetic potential, environmental factors may influence the prevalence of umbilical hernias in dairy calves. Key management aspects such as colostrum feeding, prenatal and navel cares, nutrition, calving area, and housing play crucial roles in preventing umbilical infections. Many veterinarians have observed that umbilical infections often delay umbilical closure leading to hernia [4].

The closed technique is preferred for surgeries performed under field conditions due to its ability to reduce the risk of surgical infection [16]. Similarly, Sutradhar et al. reported lower complication rates for the closed technique [19]. Conversely, the open technique is recommended for larger hernias as it allows thorough inspection of the abdominal viscera, is potentially less traumatic [5], and is associated with a low recurrence rate due to the closure of a freshly debrided abdominal wall [16]. In the present study, there was no significant difference between open and closed methods of herniorrhaphy of simple umbilical hernias. Overall, the prognosis for the surgical treatment of umbilical hernia appears favorable when diagnosed early, performed using a clean and aseptic surgical technique, and supported by effective prevention of surgical site infection during the healing period.

The present study identified umbilical abscesses as the predominant concurrent infection in calves with hernia. This suggested a possible link between specific infection types, particularly abscesses and a heightened risk of hernia formation. In light of these observations, implementing routine and meticulous umbilical cord care practices alongside ensuring proper colostrum feeding for newborn calves emerges as a crucial preventive strategy [4, 5, 23]. These measures can significantly reduce the risk of umbilical infections, potentially leading to a decrease in the prevalence of umbilical hernias, particularly in Holstein breeds raised under extensive management system where routine care might be less frequent.

The present study yielded promising outcomes of herniorrhaphy as a treatment method for uncomplicated umbilical hernias in calves. Open surgical repair also demonstrated efficacy in managing complicated hernias following successful resolution of concurrent infections through the conservative treatment protocols. The observed recurrence rates for both surgical techniques (3.49% for simple, 2.63% for complicated) suggested their potential compatibility within the framework of our current husbandry practices. However, it is imperative to engage in clear communication with farmers and livestock owners regarding the associated economic considerations. These include not only the direct medical and surgical expenses but also the potential devaluation of breeding stock afflicted with umbilical hernias. Consequently, it is recommended to designate calves that have undergone successful surgical repair for fattening and subsequent slaughter [17].

Although surgical intervention remains successful in the treatment of the hernias, however, incorporating sire selection criteria that prioritize offspring free from umbilical hernias could be a viable strategy to genetically mitigate their prevalence. Additionally, the systematic examination of all breeding stock during their calfhood for a definitively closed umbilical ring, documented by veterinary certification, can serve as a valuable preventive measure [4, 17]. Herniorrhaphy through surgical intervention is a highly effective approach for treating calves with umbilical hernias. In the present study, all calves with hernia underwent surgical treatment and there were no criteria to determine whether the hernias were of congenital or inherited origins. In this study, only the recurrence of hernia was included in data analyses. Data on other surgical complications could not be obtained due to inconsistencies in owner compliance with the recommended postsurgical local and systemic care protocols.

## 5. Conclusion

The hernia was more prevalent in younger calves (under 14 weeks old), and there was no sex association. Furthermore, the prevalence was higher in Holstein breeds raised in extensive management system. Simple umbilical hernias were the most commonly diagnosed type. A noteworthy finding was the statistically significant association between umbilical infections and an increased risk of hernia formation. No significant difference was found between the open and closed methods of herniorrhaphy of simple umbilical hernias. Surgical intervention demonstrated a favorable outcome; however, the congenitally affected calves have to be treated for fattening and slaughter.

## Figures and Tables

**Table 1:** The frequency of simple and complicated umbilical hernia in relation to age, sex, and breed in 267 calves.

Age	Sex	Breed	Sum	Simple hernia no. (%)	Complicated hernia no. (%)				
					Abscess	Enlarged stalk	UVA infection	Fistulae aplasia	Sum
≤ 30	Male	Holstein	21	16	3	1	1	—	5
		Native	5	4	1	—	—	—	1
		Others	1	1	—	—	—	—	—
	Female	Holstein	31	25	3	3	—	—	6
		Native	4	4	—	—	—	—	—
		Others	2	1	—	1	—	—	1

≥ 31–≥ 60	Male	Holstein	39	33	2	1	—	3	6
		Native	2	2	—	—	—	—	—
		Others	0	—	—	—	—	—	—
	Female	Holstein	29	26	2	—	1	—	3
		Native	5	4	1	—	—	—	1
		Others	3	3	—	—	—	—	—

≥ 61–≥ 90	Male	Holstein	27	24	2	—	1	—	3
		Native	1	1	—	—	—	—	—
		Others	0	—	—	—	—	—	—
	Female	Holstein	23	20	2	1	—	—	3
		Native	5	3	1	1	—	—	2
		Others	0	—	—	—	—	—	—

≥ 91–≥ 336	Male	Holstein	32	31	—	1	—	—	1
		Native	2	1	1	—	—	—	1
		Others	3	2	—	—	1	—	1
	Female	Holstein	27	24	1	—	1	1	3
		Native	3	2	1	—	—	—	1
		Others	2	2	—	—	—	—	—

Sum	Male	Holstein	119	104 (45.4%)	7	3	2	3	15 (39.47%)
		Native	10	8 (3.5%)	2	0	0	0	2 (5.26%)
		Others	4	3 (1.3%)	0	0	1	0	1 (2.63%)
	Female	Holstein	110	95 (41.5%)	8	4	2	1	15 (39.47%)
		Native	17	13 (5.7%)	3	1	0	0	4 (10.53%)
		Others	7	6 (2.6%)	0	1	0	0	1 (2.63%)

Sum			267	229 (85.77%)	20 (7.49%)	9 (3.37%)	5 (1.87%)	4^∗^ (1.50%)	38 (14.23%)

*Note:* Holstein: Holstein and Holstein-native cross. Others: Simmental, Holstein–Simmental cross, Simmental-native cross. UVA infection: infection of the urachus, umbilical vein, and paired umbilical arteries in separate or concurrently.

^∗^Including 2 calves with segmental aplasia.

**Table 2:** The relative frequency between umbilical hernia with umbilical infection.

Umbilical hernia	Umbilical infection		Total
	Affected—no. (%)	Nonaffected—no. (%)	
Affected	38 (15.32)	229 (9.26)	267
Nonaffected	210 (84.67)	2244 (90.74)	2454
Total	248	2473	2721

## Data Availability

The data that support this study are available from the corresponding author upon request.

## Supporting Information

Graphical Abstract Prevalence of umbilical hernia was highest in Holstein dairy calves managed under the range system.
