# Supplementary material for: A Retrospective Study on Prevalence and Surgical Management of Umbilical Hernias in Calves, 1998–2020
Source: Vet Med Int. 2025 Apr 14;2025:8838445. doi: 10.1155/vmi/8838445 (PMC12011470; doi:10.1155/vmi/8838445)
Supplement: Supporting Information — Additional supporting information can be found online in the Supporting Information section. [file 8838445.f1.pptx]

## Slide 1
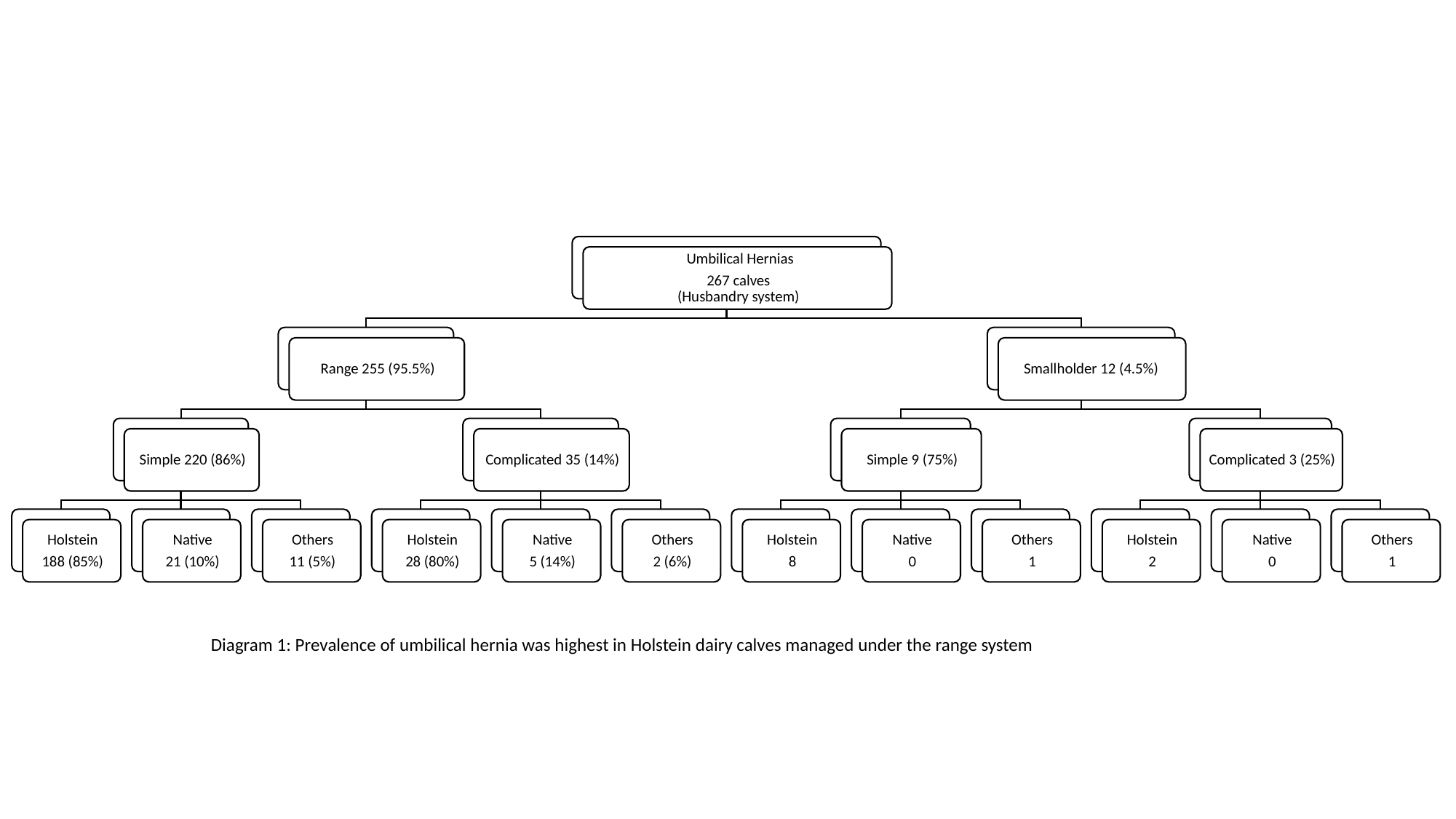

Diagram 1: Prevalence of umbilical hernia was highest in Holstein dairy calves managed under the range system
